# Oxidative stress-mediated memory impairment during aging and its therapeutic intervention by natural bioactive compounds

**DOI:** 10.3389/fnagi.2022.944697

**Published:** 2022-07-25

**Authors:** Padmanabh Singh, Bhabotosh Barman, Mahendra Kumar Thakur

**Affiliations:** ^1^Department of Zoology, Banaras Hindu University, Varanasi, India; ^2^Department of Zoology, Indira Gandhi National Tribal University, Amarkantak, India

**Keywords:** oxidative stress, aging, memory, bioactive compounds, neurodegeneration

## Abstract

Aging and associated neurodegenerative diseases are accompanied by the decline of several brain functions including cognitive abilities. Progressive deleterious changes at biochemical and physiological levels lead to the generation of oxidative stress, accumulation of protein aggregates, mitochondrial dysfunctions, loss of synaptic connections, and ultimately neurodegeneration and cognitive decline during aging. Oxidative stress that arises due to an imbalance between the rates of production and elimination of free radicles is the key factor for age-associated neurodegeneration and cognitive decline. Due to high energy demand, the brain is more susceptible to free radicals-mediated damages as they oxidize lipids, proteins, and nucleic acids, thereby causing an imbalance in the homeostasis of the aging brain. Animal, as well as human subject studies, showed that with almost no or few side effects, dietary interventions and plant-derived bioactive compounds could be beneficial to recovering the memory or delaying the onset of memory impairment. As the plant-derived bioactive compounds have antioxidative properties, several of them were used to recover the oxidative stress-mediated changes in the aging brain. In the present article, we review different aspects of oxidative stress-mediated cognitive change during aging and its therapeutic intervention by natural bioactive compounds.

## Introduction

Aging is a multifactorial complex process, characterized by progressive loss of biochemical and physiological functions, maintenance of tissue homeostasis, resistance to multiple forms of stress, and increased susceptibility to numerous diseases. This naturally occurring phenomenon is determined genetically and influenced epigenetically by the environment (Gorni and Finco, 2020; Kandlur et al., 2020). The increasing evidence of cellular, biochemical, and molecular studies showed an intimate link between the aging brain or associated neurodegenerative diseases and oxidative stress conditions. Oxidative stress denotes an imbalance between oxidants, in particular reactive oxygen species (ROS) (free radicals) and antioxidant reserve. ROS is generated in the electron transport chain (ETC) during oxidative phosphorylation in mitochondria (Grimm and Eckert, 2017; Ionescu-Tucker and Cotman, 2021). The brain mostly depends on oxidative phosphorylation to fulfill its high energy demand due to the continuous requirement of proteins, neurotransmitters synthesis, and polymerization of actin filaments. The oxidative phosphorylation-driven ROS generation is eliminated or neutralized by the antioxidant enzymes present in the brain. However, antioxidant defense systems are compromised in the aging brain, thus making it more vulnerable to ROS (Mattson et al., 2008; Srivas et al., 2020).

The “Free-radical theory of aging” stated that the damage induced in the cells by the production of intracellular free radicals is the major determinant of the aging process (Harman, 1956, 1992, 2006). These free radicals have deleterious effects on mitochondria and other subcellular organelles by promoting the oxidation of nucleic acids, proteins, and lipids, which accelerate the neurodegeneration process and ultimately lead to the age-associated decline of several cognitive functions including attention, sensory perception, decision-making, as well as learning and memory. Further, oxidative free radicals are the main predisposing factors for the transition from normal brain aging to the pathological conditions like mild cognitive impairment and neurodegenerative diseases such as Alzheimer’s disease (AD), Parkinson’s disease (PD), and Huntington’s disease (HD) (Cabral-Costa and Kowaltowski, 2020; Norat et al., 2020).

As the majority of plants have high nutritional value, they are consumed as food and medicine throughout the world. They contain several compounds that are biologically active and beneficial in different physiological and pathological conditions (Thakur et al., 2017). These biologically active compounds include polyphenols, carotenoids, phytoestrogens, biogenic amines, etc. Due to their antioxidant properties, these compounds efficiently scavenge or neutralize oxidative free radicals and thereby improve or recover underlying conditions (Fraga et al., 2019). Both animal models and clinical trial studies showed that these plant-derived bioactive compounds are neuroprotective and improve cognitive decline associated with aging and neurodegenerative diseases by reducing oxidative stress, mitochondrial dysfunction, and oxidative stress-mediated neurodegeneration (Singh et al., 2021).

## Oxidative stress in the aging brain

### Mitochondria: The key player of age-associated oxidative damage to the brain

The “free radical theory of aging” could not explain the precise subcellular location of free radical generation and its reactions, which are mainly focused on mitochondria. Therefore, the mitochondrial theory of aging was proposed later (Harman, 1972, 2006; Viña et al., 2003). Although many free radicals contribute to the age-associated oxidative burden, the majority of damage to the biological macromolecules was contributed by ROS. The major portion of the cellular ROS (95–98%) can be traced back to mitochondria, where it is generated as a byproduct of oxidative phosphorylation, a process that involves oxidation of NADH or FADH to produce energy across the inner mitochondrial membrane, which will be then used to phosphorylate the ADP. During mitochondrial electron transport, a fraction of electrons derived from NADH or FADH directly leak out of the ETC and react with oxygen or other electron acceptors to generate free radicals. Subsequent studies suggest that under physiological conditions, mitochondria from the brain or other organs convert 0.2–2% of total oxygen consumption into O_2•_– (Chance et al., 1979; Staniek and Nohl, 2000; St-Pierre et al., 2002). To detoxify these free radicals, eukaryotic cells have an antioxidant defense mechanism that includes several antioxidant enzymes such as superoxide dismutase (SOD), catalase, superoxide reductase, glutathione peroxidase, and heat shock proteins. MnSOD which is primarily present in mitochondria neutralizes the superoxide anion to yield H_2_O_2_. Further, glutathione peroxidase or catalase converts this H_2_O_2_ into H_2_O (Gemma et al., 2007). Several other small molecular weight antioxidants such as NADPH, thioredoxin, vitamin C and E also directly scavenge the ROS (Kregel and Zhang, 2007). Along with mitochondria, free radicals are also generated in multiple subcellular compartments by multiple enzymes such as NADPH oxidases within the plasma membrane, cyclooxygenases in the cytosol, lipid metabolism in the peroxisome, etc. (Lambeth, 2004).

The brain is a high energy-consuming organ and to sustain this high energy need, it almost exclusively depends on oxidative phosphorylation. As mitochondria are the primary site for oxidative phosphorylation, it is not only the major producer of ROS but also the main target of oxyradical attack (Navarro and Boveris, 2010; Grimm and Eckert, 2017). Mitochondria of the aging brain have a marked deficit in electron transfer in complex I and IV, decreased membrane potential, loss of cristae, and increased size and fragility. A reduction in the α-subunit of mitochondrial F1 ATP synthase leads to decreased ATP production in the aging brain (Navarro and Boveris, 2010; Ionescu-Tucker and Cotman, 2021). The aging brain has a compromised antioxidant defense system with reduced antioxidant enzyme activity such as SOD, catalase, and glutathione peroxidase (Gemma et al., 2007). Age-associated accumulation of mitochondrial oxidative damage also disrupts the mitochondrial dynamics by balancing its fission, fusion, and autophagy mechanisms (Lee et al., 2007; Grimm and Eckert, 2017; Mishra and Thakur, 2022). The mitochondrial DNA (mtDNA) is also highly susceptible to oxidative stress-mediated damages (Richter et al., 1988; Gemma et al., 2007). Neuron as a post-mitotic cell seems to be more sensitive to mitochondrial dysfunction-induced oxidative damages as compared to dividing cells (Kowald and Kirkwood, 2000; Terman et al., 2010). Overall, these studies suggest that age-associated increase in free radical generation leads to damage of mitochondrial lipids, proteins, and DNA which ultimately causes mitochondrial dysfunction and thus plays a key role in age-associated changes in the brain (Paradies et al., 2011).

### Lipid peroxidation

The peroxidation of biological lipids is one of the major outcomes of oxidative stress-mediated tissue damage. The brain has an abundance of unsaturated lipids which make it highly susceptible to peroxidation and oxidative modifications. Free radical-mediated lipid peroxidation can directly damage the cellular membranes or generate several highly reactive secondary products such as malondialdehyde (MDA), 4-hydroxy-2 hexenal (HHE), 4-hydroxyl-2-non-enal (HNE), acrolein, isoprostanes, neuroprostanes through fission and endocyclization of oxygenated fatty acids (Floyd and Hensley, 2002; Montine et al., 2002; Gemma et al., 2007; Paradies et al., 2011). Sensitivity to oxidation also increases exponentially with the number of double bonds per unsaturated fatty acids. Unsaturated phospholipids such as polyunsaturated fatty acid (PUFA) are the major components of plasma, and mitochondrial membranes and are abundant in the aging brain. Peroxidation of membrane lipid disrupts the organization of lipid bilayer and thus alters the membrane fluidity and permeability, lipid-lipid and lipid-protein interaction dynamics, ion and nutrient transport, membrane potential, membrane receptor-mediated signaling pathway, and activity of membrane-bound enzymes (Pamplona, 2008; Paradies et al., 2011; Catalá and Díaz, 2016). Cardiolipin, a phospholipid that is exclusively localized within the inner mitochondrial membrane, plays a key role in mitochondrial membrane organization and several bioenergetic processes. Free radical-mediated oxidation/depletion of cardiolipins causes mitochondrial dysfunction during aging (Pamplona, 2008; Paradies et al., 2011).

Aldehydes and isoprostanes produced from lipid peroxidation are biologically active. The aged brain of several organisms including humans shows an increased level of MDA, HNE, and isoprostanes (Yoritaka et al., 1996; Dei et al., 2002; Zarkovic, 2003). HNE forms covalent adducts with histidine, lysine, and cysteine residues of the proteins and causes the inactivation of several enzymes such as glutathione peroxidase and α-ketoglutarate dehydrogenase. Excessive production of HNE disrupts Ca^2+^ ion homeostasis, impairment of glucose and glutamate transport, suppression of NFkB activity, and activates the caspase pathway (Floyd and Hensley, 2002; Sultana et al., 2013). MDA reacts with several DNA bases such as deoxyguanosine, deoxyadenosine and generates exocyclic DNA adducts which ultimately disrupt the DNA base-pairing (Gemma et al., 2007; Gentile et al., 2017). MDA also forms adducts with proteins and causes age-associated accumulation of lipofuscin, an intralysosomal fluorescent pigment in different tissues including the brain (Pamplona, 2008; Jové et al., 2019). Lipid peroxidation mediated damage to the α and β subunit of the ATP synthase causes inactivation of the complex and deficits in ATP synthesis (Jové et al., 2019).

### Protein oxidation

Accumulation of oxidized and modified proteins is one of the hallmarks of the aging brain. Along with other multitudes of modification events (e.g., faulty post-translational modification, halogenation, glycation, deamidation, racemization), reactive oxygen or nitrogen species causes changes in both protein backbone and amino acid side chains. Oxidative radical-induced damage to the protein backbone results in the formation of peroxyl radicals which ultimately cause fragmentation of the protein backbone (Stadtman, 1992, 2004; Grimm et al., 2011; Reeg and Grune, 2015). Oxidation of amino acid side chains leads to the generation of several oxidation products. Some widely studied protein oxidation products in the brain include methionine sulfoxide, di-tyrosine, ortho-tyrosine, formation of disulfide bonds at cysteine residues, and carbonyl derivatives. The expression of protein carbonyls, ortho-tyrosine, and methionine sulfoxide shows a logarithmic increase in several regions of the brain with age (Davies et al., 1987; Dalle-Donne et al., 2003; Kregel and Zhang, 2007; Reeg and Grune, 2015). Oxidation of proteins causes a partial unfolding of their structure and thus impairs their function. Most amino acid residues can be oxidized, though histidine, methionine, and cysteine are more sensitive. Oxidative modifications of those amino acid residues lead to the impairment of the catalytic activity of several enzymes such as protein tyrosine phosphatases, glutamine synthetase, and creatine kinase. Oxidation of a methionine residue of calmodulin causes the loss of its calcium-binding ability, responsible for the activation of several calcium-sensitive enzymes and ion channels (Smith et al., 1991; Kregel and Zhang, 2007; Drazic and Winter, 2014; Reeg and Grune, 2015). ROS modulates the DNA binding activity of several transcription factors such as NFkB, forkhead transcription factor, activator protein-1 (AP-1), and p53 either by direct modification of their critical amino acid residues or *via* indirect regulation of their phosphorylation/dephosphorylation (Kregel and Zhang, 2007).

Cellular proteolytic mechanisms (proteasomal and autophagic pathways) are responsible for the degradation of most oxidized, aggregated, and misfolded proteins. Both 20S and 26S proteasomes (ubiquitin-proteasome system or UPS) play an important role in the removal of oxidized proteins and are dysregulated in the aging brain and other neurodegenerative diseases. Besides the proteasomal degradation system, the autophagy-lysosomal pathway is also responsible for clearing misfolded proteins. Recent studies suggest the age-associated downregulation in macroautophagy and chaperone-mediated autophagy (CMA) in the brain (Reeg and Grune, 2015; Zheng et al., 2016; Loeffler, 2019; Kelmer Sacramento et al., 2020). 26S proteasome is found to be more vulnerable to oxidative stress-mediated damages than the 20S proteasome. Oxidative free radicals disrupt the integrity of 26S proteasome and result in dissociation of 20S core from 19S regulator. Oxidative stress-mediated dysregulation in proteasome gene expression and post-translational modification of proteasome subunits have also been reported. The 19S and 20S subunits are susceptible to carbonylation and HNE modification which contribute to the suppression of their proteolytic activity (Aiken et al., 2011; Reeg and Grune, 2015). Thus, age-associated extensive oxidative modification of proteins and progressive loss of proteasomal degradation activity favor the accumulation of oxidized proteins in the aging brain (Farout and Friguet, 2006; Grimm et al., 2011). All these events have led to the concept of a vicious cycle: increased ROS leads to protein oxidation which may act as an endogenous inhibitor of proteasomal activity and causes the accumulation of damaged proteins that, in turn, facilitates further oxidative stress.

### DNA damage and epigenetic modifications

DNA bases are prone to oxidative stressors through direct or epigenetic manipulations. Direct oxidative damage to DNA leads to the generation of oxidized purines or pyrimidines and DNA breaks (Floyd and Hensley, 2002; Ionescu-Tucker and Cotman, 2021). Superoxide anions and hydroxyl radicals mediated oxidization of adenine, guanine, or cytosine residues causes the generation of 8-hydroxyadenine, 5-diamino-4H-imidazolone, 8-oxo-2-deoxyguanosine (oxo^8^dG), 8-hydroxy-2-deoxyguanosine (8-OHdG), 8-oxo-7,8-dihydroguanine (8-oxoG), 5-hydroxy-5,6-dihydrocytos-6-yl and 6-hydroxy-5,6-dihydrocytos-5-yl (Kandlur et al., 2020). Oxidative stress-mediated DNA damages have been commonly assessed through the presence of oxo^8^dG and 8-OHdG, both of which show a manyfold increase in both mitochondrial and nuclear DNA in normal aging and AD-associated brain tissue (Floyd and Hensley, 2002; Balaban et al., 2005; Mecocci et al., 2018). The mtDNA contains manyfold higher damaged DNA as compared to nuclear DNA. Such high susceptibility of mtDNA to oxidative stress might be due to its proximity to a primary source of ROS, absence of protective histone covering, and deficient repair mechanisms (Balaban et al., 2005; Kregel and Zhang, 2007). DNA breaks can be both single or double-stranded. As double-stranded breaks near the gene promoter can change the gene transcription, it is more toxic than single-strand breaks (Shanbhag et al., 2019; Ionescu-Tucker and Cotman, 2021). During normal aging or AD, there are deficits in the base excision repair pathway, which ultimately causes age-related accumulation of double as well as single-strand DNA breaks in the brain tissue (Rutten et al., 2007; Thanan et al., 2014).

Oxidative stress directly modulates the epigenetic modification of chromatin by changing DNA methylation and post-translational histone modification. It downregulates the DNA methylation by increasing TET-mediated hydroxymethylation, interfering binding of DNA methyltransferases, and oxidizing 5-methylcytosine (5mC) or cytosine (Chia et al., 2011; Thanan et al., 2014). This shows a positive correlation with the downregulation of global methylation in the aging brain. ROS-mediated alteration of histone acetylation and methylation can change the chromatin structure, gene stability, and expression. The emerging evidence suggests that oxidative stress-mediated upregulation of H3K9 methylation might cause cognitive aging and AD pathology (Gu et al., 2013; Kandlur et al., 2020; Ionescu-Tucker and Cotman, 2021).

### Oxidative stress-mediated memory impairment during aging

Several cellular, molecular and behavioral studies suggest accumulated oxidative stress as one of the main causal factors involved in the initiation and progression of cognitive deficits during aging or age-associated neurodegenerative diseases (Mecocci et al., 2018; Kandlur et al., 2020). The hippocampus and frontal lobe of the brain are more prone to oxidative stress-induced damages, which suggests an intimate link between the age-associated decline of cognitive abilities such as information acquisition, retrieval of declarative memories, attention, and language skill with increased oxidative stress (Nagai et al., 2003; Rodrigues Siqueira et al., 2005; Castelli et al., 2019). This phenomenon is supported by studies that the overexpression of antioxidant enzymes or antioxidants supplementation in aging animals showed alleviation of spatial learning memory, working memory, and increased consolidation/retention capacity, and vice versa young animals subjected to oxidative stress showed a decline in memory function as found in normal aging (Fukui et al., 2002; Hu et al., 2006; Head, 2009; Haddadi et al., 2014). Age-associated memory impairment is believed to be a consequence of the functional deterioration of neurons and their degeneration. Oxidative stress damages nerve terminals by mitochondrial dysfunctioning, abnormal accumulation of synaptic vesicles, and decline in neurotransmitter release which ultimately induce a deficit of synaptic membrane depolarization and cause deterioration of the neurotransmission system (Fukui et al., 2002; Kamat et al., 2016). These abnormalities are also commonly found in the aging brain. Downregulated synaptic plasticity-related immediate-early gene expression also plays a major role in age-associated cognitive impairment (Barman et al., 2021). Oxidative stress-mediated unrepaired DNA damage at those genes promoters causes downregulation of their expression (Ionescu-Tucker and Cotman, 2021). Due to their post-mitotic nature, neurons are non-dividing cells which makes them especially prone to oxidative stress-mediated aggregation of modified proteins or other biological macromolecules, robust mitochondrial dysfunctioning that ultimately leads to neuronal cell death or neurodegeneration (Andersen, 2004; Grimm et al., 2011). Another main contributing factor to age-associated cognitive dysfunction is oxidative stress-mediated cellular senescence, which causes an overall decrease in brain weight and volume and expansion of cerebral ventricles (Haddadi et al., 2014; Mecocci et al., 2018; Pesce et al., 2018).

## Natural bioactive compounds and their sources

**FIGURE 1:**
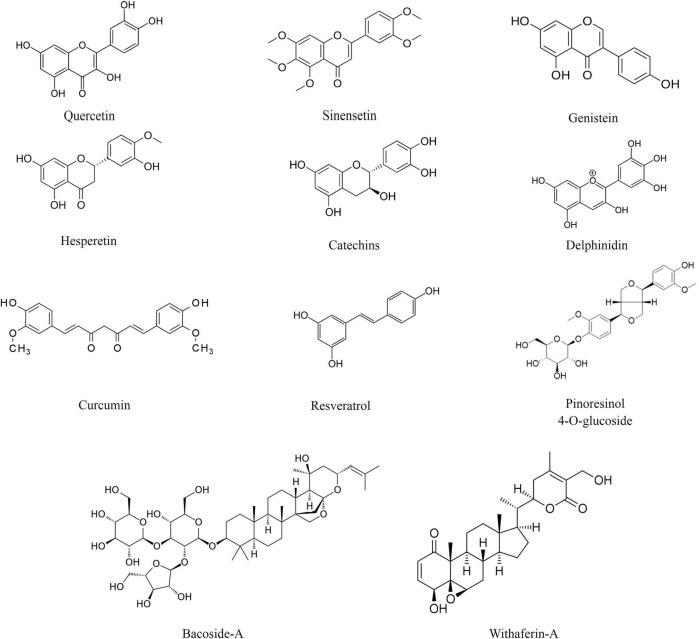
Chemical structure of selected plant-derived bioactive compounds.

Plant-derived bioactive compounds are widely used as an antioxidant. The most commonly used molecules as antioxidants are plant-derived polyphenols, i.e., resveratrol, curcumin, genistein, epigallocatechin gallate, etc. Polyphenols are plant-derived secondary metabolites synthesized from the shikimate or polyketide pathway and commonly found in fruits, vegetables, seeds, and nuts. Polyphenols contain multiple phenols units (C_6_H_5_OH) where the hydroxyl group (OH) is attached to the aromatic benzene ring (Quideau et al., 2011; Figure 1). Based on their composition and number of phenol subunits, polyphenols may be divided into flavonoids, phenolic acids, stilbenes, and lignans (Brglez Mojzer et al., 2016; Table 1). They have not only antioxidative properties, but these secondary metabolites provide color and aroma to fruits and vegetables. Apart from phenolic compounds, non-phenolic compounds also play an important role in alleviating oxidative stress and improving memory in animal models as well as human subjects (el-Sayed et al., 2008).

**TABLE 1:** Major plant-derived bioactive compounds and their sources.

Bioactive compounds			Examples	Sources
Plant polyphenols	Flavonoids	Flavonols	Quercetin, Kaempferol	Cucumber, Broccoli, Strawberries
		Flavones	Sinensetin, Tangeretin	Parsley, Orange, Citrus peels
		Isoflavones	Genistein, Daidzein	Soy, Soy-based products
		Flavanones	Hesperetin, Narirutin	Orange, Lemon
		Flavanols	Catechins, Proanthocyanidins	Apple, Apricot, Blueberry
		Anthocyanins	Delphinidin, Peonidin	Cherry, Peach, Plum, Cranberry
	Phenolic acids		Curcumin, Gallic acid	Turmeric, Berries, Tea, Cereal
	Stilbenes		Resveratrol, Diethylstilbestrol	Grapes, Berries
	Lignans		Pinoresinol 4-*O*-β-D-glucopyranoside	Seeds, Cereals
Non-phenolic compounds			Bacoside-A, Withaferin-A, Withanolide-A	Brahmi, Ashwagandha

### Flavonoids

Flavonoids are major groups of polyphenols that contain two aromatic rings (ring A and ring B) and one heterocyclic ring (ring C). They are further classified into six different groups- flavonols, flavones, isoflavones, flavanones, flavanols, and anthocyanins. In flavonols, the heterocyclic ring C contains a ketone group and a hydroxyl group. Examples of flavonols are fisetin, quercetin, and kaempferol. The major source of flavonols includes kale, cucumber, broccoli, blueberries, strawberries, onions, etc. (Tsao, 2010). In flavones, the heterocyclic ring C contains a double bond and a ketone group. Examples of flavones are sinensetin and tangeretin. Wheat, parsley, orange, citrus peels, mandarins, etc., are rich sources of flavones (Manach et al., 2004). The isoflavones are also known as phytoestrogens as they show structural similarities with the steroid hormone estrogen. Examples of isoflavones are genistein and daidzein. The main source of isoflavones includes soy and soy-based food such as milk, tofu, and beverages (Rasouli et al., 2017). In flavanones, the heterocyclic ring C is fully saturated. Examples of flavanones are hesperetin and narirutin. Flavanones are mainly found in citrus fruits such as orange and lemon. In flavanols, the heterocyclic ring C contains a hydroxyl group. Examples of flavanols are catechins and proanthocyanidins. The major dietary sources of flavanols are blueberries, red wine, apples, apricots, etc. (Quideau et al., 2011). Anthocyanins contain a three-carbon positively charged oxygenated heterocyclic C ring. They are plant pigments and provide color to flowers and fruits. Examples of anthocyanins are delphinidin, pelargonidin, peonidin, and malvidin. The major source includes fruits and flowers of higher plants (Mazza and Miniati, 1993; Table 1).

### Phenolic acids

Phenolic acids are aromatic polyphenols that contain phenolic rings and carboxylic acid. They may be hydroxybenzoic acids (derived from benzoic acid) and hydroxycinnamic acids (derived from cinnamic acid) (Quideau et al., 2011). Examples of phenolic acids are curcumin, ellagitannins, gallic acid, ferulic acid, etc. The dietary source of phenolic acids is strawberries, blackberries, turmeric, tea, cereals, and grains (Hano and Tungmunnithum, 2020).

### Stilbenes

Stilbenes are a group of polyphenols that are synthesized in low quantities as a result of injury or infection due to microorganisms. They contain two aromatic phenolic rings connected by a methylene group (Quideau et al., 2011). Examples of stilbenes are resveratrol and diethylstilbestrol. The major dietary source is grapes, berries, and red wines (Cvejic et al., 2010).

### Lignans

Lignans are polyphenols that contain two phenylpropane units. They are processed by gut microbes into beneficial metabolites such as enterolactone and enterodiol. Examples of lignans are pinoresinol 4-*O*-β-D-glucopyranoside, secoisolariciresinol, and matairesinol. Cereals, seeds, fruits, and vegetables are rich sources of lignans (Sun et al., 2014).

### Non-phenolic compounds

Non-phenolic bioactive compounds are also plant-derived secondary metabolites. Their examples are bacoside-A isolated from *Bacopa monnieri* (Brahmi), withaferin-A, and withanolide-A isolated from *Withania somnifera* (Ashwagandha), and ginkgolide-B and bilobalide isolated from *Ginkgo biloba*. Brahmi and ashwagandha have been widely used in Indian ayurvedic medicine as nootropics. Similar to phenolic compounds, non-phenolic compounds are potent antioxidants (Foti and Amorati, 2009).

## Antioxidative properties of natural bioactive compounds

The antioxidant properties of bioactive compounds are contributed in two ways. First, the structures of the bioactive compounds are responsible for the scavenging of free radicals. Second, these compounds alter the expression and activities of enzymes responsible for oxidative stress metabolism. These plant-derived bioactive compounds decrease the synthesis of free radical species and thereby reduce the oxidation of biomolecules in the cells.

### Free radical scavenging properties

Due to the presence of ring structure, conjugated double bonds, and hydroxyl (OH) groups associated with the unsaturated carbon (C = C) atoms in their skeleton, plant bioactive compounds scavenge electrons and free radicals generated during oxidative stress (Figure 1). These structures scavenge or neutralize oxygen free radicals by donating the hydrogen (H) atom or an electron from the hydroxyl group or by delocalizing an unpaired electron in the conjugated aromatic ring (Dai and Mumper, 2010). Genistein is a plant-derived phytoestrogen belonging to the group of isoflavones. It has been widely used as an antioxidant in the recovery of oxidative stress. The free radical scavenging properties of genistein are shown by the A, B, and C-ring as well as the double bond (C-2,3) in association with the functional group (4-oxo). The B and C-ring, double bond along with the 4-oxo functional group delocalize the electrons from the free radicals and neutralize them (Bors et al., 1990). Apart from delocalizing electrons from free radicals, the B-ring also forms stable compounds after reacting with the free radicals. The B-ring reacts with the peroxyl free radicals to form stable compounds like orobol and 4′-oxogenistein (Arora et al., 2000). Hydroxyl (–OH) groups on A-ring (C-5′ and C-7′ positions) and B-ring (C-4′ position) inhibit the oxidation of low-density lipoproteins or lipid peroxidation (de Whalley et al., 1990; Chen et al., 1996; Arora et al., 1998).

Polyphenol curcumin is an important plant-derived compound that shows antioxidant properties. Similar to genistein, the structure of curcumin plays an important role in quenching or scavenging free radicals and ROS. The antioxidant property of curcumin is due to the presence of β-diketone structure and two hydroxyl groups present at the ortho position of the aromatic ring (Jovanovic et al., 1999; Malik and Mukherjee, 2014). Further, the hydroxyl group along with the electron releasing ability of the methoxy group (–OCH_3_) is also important for the scavenging of free radicals (Somparn et al., 2007).

Resveratrol (3,5,4′-trihydroxy-trans-stilbene) is a phytochemical mainly found in the grapes and berries such as blueberries and raspberries. It contains two aromatic rings and three hydroxyl groups (two meta and one para). The antioxidant properties of resveratrol are due to the chelating as well as scavenging action. It chelates metal ions such as zinc, iron, copper, and aluminum and scavenges oxygen free radicals (Leonard et al., 2003; Gülçin, 2010). Stojanović et al. (2001) reported that the para hydroxyl group shows better free radical scavenging properties than the meta-hydroxyl groups. Gallic acid (3,4,5-trihydroxy benzoic acid) is a phenolic acid found in the tea leaves and bark of the oak tree. Similarly, the free radical scavenging properties of gallic acid are due to the presence of three hydroxyl groups and the aromatic ring (Rajan and Muraleedharan, 2017; Zeb, 2020). Quercetin (3,3′,4′,5,7-Pentahydroxyflavone) is a flavonol mostly found in the fruits and vegetables such as red onion and kale. Due to the presence of five aromatic hydroxyl groups, quercetin shows higher antioxidant and free radical scavenging properties (Tsao, 2010). Mukherjee et al. (2011) analyzed the free radical scavenging properties of Brahmi. FRAP assay, DPPH free-radical scavenging assay, reducing power assay, and lipid peroxidation assay showed that the aqueous and ethanolic extracts of Brahmi efficiently scavenge free radicals.

### Regulation of the activity and expression of antioxidant enzymes

Apart from direct quenching or scavenging oxidative free radicals, plant-derived bioactive compounds also alter the expression of pro and antioxidant enzymes in different physiological conditions. The pro-oxidant enzyme and metabolites are monoamine oxidase (MAO) and malondialdehyde, and the antioxidant enzymes are catalase, SOD, and glutathione peroxidase.

Previous research on animal models shows that isoflavone genistein alters the pro and antioxidant enzyme system and thus decreases oxidative stress. Chronic administration of genistein (15 and 30 mg/kg) decreased the level of fatty acid oxidation product malondialdehyde and the activity of prooxidant enzyme MAO and increased the activity of the antioxidant enzyme in the cortex and hippocampus of ovariectomized (OVX) rats. Further, change in malondialdehyde level and MAO and SOD activity lowered lipid peroxidation and improved spatial memory in OVX rats (Huang and Zhang, 2010). Scopolamine is a muscarinic receptor antagonist which increases the ROS level and induces amnesia (Konar et al., 2011; Singh et al., 2015). Lu et al. (2018) reported that genistein pretreatment showed beneficial effects on scopolamine-induced amnesic mice. Scopolamine increased malondialdehyde level and decreased glutathione content and SOD activity in the hippocampus of control mice as compared to the genistein pretreated mice. Similarly, Qian et al. (2015) also reported that genistein pretreatment decreased ROS generation, caspase-9/3 activities, and neuronal death in H_2_O_2_-treated cortical neuronal culture. Resveratrol is a well-studied stilbene and showed a beneficial effect in reducing oxidative stress in animal models as well as in cell culture experiments. Pretreatment of resveratrol increased the activities of antioxidant enzymes glutathione peroxidase, catalase, and SOD and reduced lipid peroxidation and cerebral edema in hypoxic-ischemic induced brain injury in neonatal rats (Gao et al., 2018). In a similar study, Wang et al. (2014) reported that pretreatment of resveratrol increased the SOD activity and reduced MDA levels in the hippocampus due to cerebral ischemia in rats. Resveratrol pretreatment also reduced mitochondrial damage and protected neurons from apoptosis in cerebral ischemia-induced rats.

The phenolic acid curcumin is widely consumed as a spice and herbal medicine in India and around the world. Studies showed that curcumin modulates the expression and activity of antioxidant enzymes in different animal models. Curcumin supplementation increased the mean life span of *Drosophila* and was associated with higher SOD enzyme activity (Suckow and Suckow, 2006). Similarly, curcumin treatment increased the SOD and glutathione peroxidase activity and decreased lipid peroxidation in different brain regions such as the cortex, hippocampus, and cerebellum in 24-month-old rats (Bala et al., 2006). Youssef et al. (2020) examined the antioxidant properties of pinoresinol-4-*O*-β-D-glucopyranoside (PGu), a lignan isolated from the plant *Prunus domestica*, in a lithium/pilocarpine-induced rat model of epilepsy. They observed that PGu decreased the malondialdehyde level and increased the activity of catalase enzyme in the cerebral cortex and thus reducing seizures in the epileptic rats.

## Role of natural bioactive compounds in the recovery of age-associated memory impairment

Natural bioactive compounds have been extensively used to improve or restore learning and memory in different animal models. Such memory-enhancing or restoring properties of bioactive compounds are achieved by reducing oxidative stress as well as by decreasing the oxidative stress-mediated mitochondrial dysfunctions, oxidation of biomolecules, and neurodegeneration during aging and age-associated neurodegenerative diseases. Antioxidative properties of plant-derived bioactive compounds have been studied to recover or improve learning and memory during aging as well as during neurodegenerative diseases both in human subjects and animal models.

AD is a neurodegenerative disease characterized by accumulation of amyloid-beta (Aβ) peptide and neuronal cell death leading to impairment of cognitive functions. The soy isoflavone genistein is used both in physiological aging and in neurodegenerative disease mouse models. Studies show that genistein can easily cross the blood-brain barrier (Tsai, 2005; Pierzynowska et al., 2018). Bagheri et al. (2011) examined the antioxidant properties of genistein on an Aβ (1–40) induced AD rat model, which shows learning and memory impairment in the Y maze task, passive avoidance task, and RAM task. Further, genistein treatment decreased malondialdehyde levels, increased SOD activity, and improved learning and memory in an AD rat model. Similar to Aβ (1–40), Aβ (25–35) also increased oxidative stress and induced neuronal cell death. In an *in vitro* neuronal cell culture study, Aβ (25–35) increased accumulation of oxygen free radicals, intracellular Ca^2+^ level, and nuclear DNA damage. Further, administration of genistein showed neuroprotection by reversing the effects of Aβ (25–35) on cell culture (Zeng et al., 2004). HD is a neurodegenerative disease characterized by the aggregation of mutant HTT gene products in the neurons which leads to neurodegeneration, motor abnormalities, and cognitive dysfunction. 3-Nitropropionic acid (NPA), a mycotoxin and inhibitor of mitochondrial respiratory complex II, is used to induce HD phenotypes in animal models (Túnez et al., 2010). Administration of NPA increased oxidative stress, expression of cycloxygenase-2, inducible nitric oxide synthase (iNOS), and impaired cognitive functions. Further, pretreatment of genistein improved cognitive functions, decreased oxidative stress, and expression of cycloxygenase-2 and iNOS in the cortex and hippocampus of OVX rats (Menze et al., 2015). Lu et al. (2018) reported that genistein pretreatment showed beneficial effects on scopolamine-induced amnesic mice. Genistein treatment increased the expression of memory-linked genes p-ERK, p-CREB, and BDNF in the hippocampus of scopolamine-induced amnesic mice.

The polyphenolic compounds resveratrol and its derivatives such as piceatannol, pterostilbene, and scirpusin A showed neuroprotective effects in aging and AD animal models (Rege et al., 2014). Navarro-Cruz et al. (2017) examined the antioxidant effects of resveratrol on the cognitive performance of aging rats. They observed that chronic administration of resveratrol reduced nitrite and malondialdehyde levels in the brain and increased hippocampal-dependent recognition memory during aging. Huang et al. (2011) reported that Aβ peptide increased iNOS expression, lipid peroxidation in the hippocampus, and impaired spatial memory in rats. Resveratrol administration decreased Aβ peptide accumulation, reversed iNOS expression, and lipid peroxidation, and improved spatial memory in AD rat models. Microtubule-disrupting compound colchicine causes loss of cholinergic neurons and cognitive dysfunction similar to the phenotypes observed in AD animal models. Intracerebroventricular colchicine administration increased free radical generation, decreased glutathione activity in the rat brain, and impaired memory performance in Morris water maze and elevated plus-maze task. Chronic resveratrol treatment decreased free radical generation and improved memory in colchicine-treated rats (Kumar et al., 2007).

Earlier studies showed that curcumin effectively attenuated the oxidative stress-mediated cognitive impairment in different animal models. Apart from normal physiological aging, chemically induced (i.e., D-galactose) aging or senescence model has been widely used to mimic the age-associated changes. Chronic high dose intake of reducing sugar D-galactose induced oxidative stress and neuronal cell death. Lee et al. (2020) reported that D-galactose administration increased oxidative stress, apoptosis, and expression of senescence marker proteins such as p16 and p21. Supplementation of plant flavonoids curcumin and hesperetin increased the expression of antioxidant enzymes, decreased oxidative stress, apoptosis, and the expression of p16 and p21. Further, supplementation of curcumin and hesperetin alone or in combination improved cognitive impairment due to D-galactose. Aluminum chloride (AlCl_3_) treated animal models are used to study the sporadic form of AD. The model is characterized by high oxidative stress, decreased antioxidant enzyme activities, neuronal cell death, and neurodegeneration. Supplementation of curcumin significantly decreased MDA level and increased the activity of SOD and catalase enzyme in the hippocampus of AD rats. Further, curcumin attenuated the neurodegeneration and decline of recognition memory in the AlCl_3_-treated AD rats (ELBini-Dhouib et al., 2021; Table 2).

**TABLE 2:** Antioxidative properties of plant-derived bioactive compounds and their involvement in memory recovery.

Bioactive compounds	Antioxidative properties and role in memory recovery	References
Flavonols (Quercetin, Kaempferol)	Scavenging free radicals including ROS; improvement in memory recall during contextual fear conditioning; improvement in spatial memory task (e.g., Morris water maze test)	Tsao, 2010; Nakagawa et al., 2016; Molaei et al., 2020
Isoflavones (Genistein, Daidzein)	Scavenging oxidative free radicals; decreased malondialdehyde and glutathione level; decreased activity of MAO; decreased ROS generation, caspase-9/3 activities, and Aβ induced neuronal death; increased activity of glutathione peroxidase, SOD and catalase; improvement in spatial memory task, Y maze task, passive avoidance task, and RAM task	Bors et al., 1990; Huang and Zhang, 2010; Bagheri et al., 2011; Qian et al., 2015; Lu et al., 2018
Flavanones (Hesperetin, Narirutin)	Increased expression of antioxidant enzymes; decreased oxidative stress and apoptosis; improved cognitive functions	Lee et al., 2020
Phenolic acids (Curcumin, Gallic acid)	Scavenging free radicals and ROS; increased glutathione peroxidase and SOD activity; decreased lipid peroxidation; decreased neurodegeneration; improvement in spatial learning; slowdown of age-associated cognitive decline and improved recognition memory	Bala et al., 2006; Suckow and Suckow, 2006; Malik and Mukherjee, 2014; Sarker and Franks, 2018; ELBini-Dhouib et al., 2021
Stilbenes (Resveratrol, Diethylstilbestrol)	Scavenging free radicals, ROS and chelates metal ions; reduced mitochondrial damage; protect neurons from apoptosis; increased SOD activity and reduced MDA level; improved learning and memory in Morris water maze and elevated plus-maze task	Leonard et al., 2003; Gülçin, 2010; Huang et al., 2011; Wang et al., 2014
Lignans (Pinoresinol 4-*O*-glucoside)	Decreased MDA level and increased catalase activity; restore impaired memory in Morris water maze and Y maze test	Youssef et al., 2020; Lei et al., 2021
Non-phenolic compounds (Bacoside-A, Withaferin-A, Withanolide-A)	Scavenging free radicals; decreased lipid peroxidation; increased activity of SOD, catalase and glutathione peroxidase; reduction in the basal level of ROS, MDA, hydroperoxides; decreased neurodegeneration; improved cognitive behavior, spatial memory and motor learning	Mukherjee et al., 2011; Shalini et al., 2021; Ben Bakrim et al., 2022

## Conclusion and prospects

**FIGURE 2:**
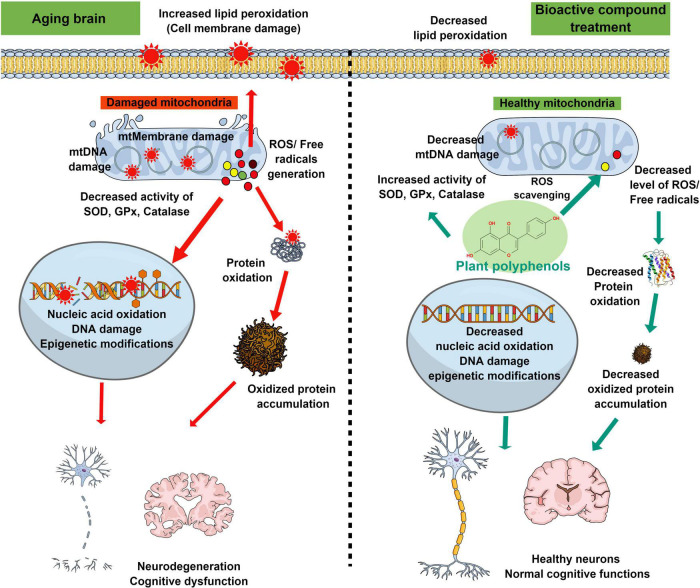
Schematic diagram representing the antioxidant properties of plant-derived bioactive compounds in the recovery of oxidative stress-mediated changes in the aging brain. Age-associated cellular and biochemical changes such as mitochondrial dysfunction, and decline of antioxidant enzyme systems lead to the generation of excessive reactive free radicals. These free radicals cause changes in the biological macromolecules (lipid peroxidation, oxidation of protein, and nucleic acid) and thus facilitate cellular or mitochondrial membrane damage, modified protein accumulation, double-stranded DNA break, and epigenetic modifications. These oxidative stress-mediated damages ultimately lead to neurodegeneration and decline of cognitive functions during aging or associated neurodegenerative diseases. Supplementation of plant-derived bioactive compounds ameliorates the age-associated neurodegeneration and cognitive dysfunctions by increasing the activities of antioxidant enzymes and decreasing the oxidative stress-induced damages to biological macromolecules.

Several studies showed that the neuroprotective and memory-enhancing activities of bioactive compounds are mediated through alleviating mitochondrial dysfunction, oxidative damage of carbohydrates, proteins, fats, and nucleic acids, and altering the expression and activity of the pro and antioxidant enzymes in the brain (Figure 2). Though these bioactive compounds have high therapeutic potential and fewer side effects, most of these molecules are still in the preclinical phase due to the absence of in-depth studies on bioavailability, metabolism, and blood-brain barrier permeability. In natural conditions, bioactive compounds are present in a stable medium and surrounded by other biomolecules. Upon isolation, extraction, and processing, the activities of the compounds get reduced or lost. Further, storage (pH, temperature, light, etc.) as well as physiological changes, and alteration of pH during digestion and metabolism by gut microbes also change the activity of these compounds. The use of green extraction technologies improves the extraction of particular compounds from fruits and vegetables and limits the use of toxic solvents (Soquetta et al., 2018; Giaconia et al., 2020). Several studies show that after ingestion, these bioactive molecules (i.e., quercetin, glycosides, and aglycone) are metabolized by enzymes in the digestive tract which affects their bioavailability (Oracz et al., 2020). Apart from digestive enzymes, gut microbes also metabolize bioactive compounds and affect their bioavailability. Gut microbes metabolize isoflavone daidzein into a more effective equol molecule (Setchell et al., 2002). The passage of a molecule through the blood-brain barrier depends on its chemical nature, molecular size, and charge on the molecule, due to which several bioactive compounds are unable to cross the blood-brain barrier. Nano-based encapsulation and drug delivery systems are very useful to increase the shelf life of these compounds as well as their solubility, stability, and bioavailability (Upadhyay, 2014). Nano-based formulation increased the solubility and antioxidant activity of kaempferol as compared to normal kaempferol molecules (Tzeng et al., 2011). PLGA nanoencapsulation increased the half-life and bioavailability of curcumin to 5.6-fold as compared to free curcumin (EFSA Scientific Committee et al., 2011). Several bioactive compounds such as resveratrol, quercetin, curcumin, etc. crossed the blood-brain barrier in very low concentrations. Nanophytomedicine derived from these bioactive compounds showed increased blood-brain barrier permeability and effectively attenuated oxidative stress in pathological conditions like PD (Ganesan et al., 2015). Furthermore, in-depth clinical studies are needed for the future use of those bioactive compounds as therapeutic agents.

## Author contributions

PS and BB prepared the manuscript and figures. MKT supervised the study and edited the manuscript. All authors participated in the design of the study and approved the submitted version.

## References

[B1] Aiken C. T. Kaake R. M. Wang X. Huang L. (2011). Oxidative stress-mediated regulation of proteasome complexes. *Mol. Cell. Proteomics* 10:R110.006924. 10.1074/mcp.M110.006924 21543789 PMC3098605

[B2] Andersen J. K. (2004). Oxidative stress in neurodegeneration: cause or consequence? *Nat. Med.* 10 S18–S25. 10.1038/nrn1434 15298006

[B3] Arora A. Nair M. G. Strasburg G. M. (1998). Structure-activity relationships for antioxidant activities of a series of flavonoids in a liposomal system. *Free Radic. Biol. Med.* 24 1355–1363. 10.1016/s0891-5849(97)00458-9 9641252

[B4] Arora A. Valcic S. Cornejo S. Nair M. G. Timmermann B. N. Liebler D. C. (2000). Reactions of genistein with alkylperoxyl radicals. *Chem. Res. Toxicol.* 13 638–645. 10.1021/tx000015a 10898596

[B5] Bagheri M. Joghataei M. T. Mohseni S. Roghani M. (2011). Genistein ameliorates learning and memory deficits in amyloid β (1-40) rat model of Alzheimer’s disease. *Neurobiol. Learn. Mem.* 95 270–276. 10.1016/j.nlm.2010.12.001 21144907

[B6] Bala K. Tripathy B. C. Sharma D. (2006). Neuroprotective and anti-ageing effects of curcumin in aged rat brain regions. *Biogerontology* 7 81–89. 10.1007/s10522-006-6495-x 16802111

[B7] Balaban R. S. Nemoto S. Finkel T. (2005). Mitochondria, oxidants, and aging. *Cell* 120 483–495. 10.1016/j.cell.2005.02.001 15734681

[B8] Barman B. Kushwaha A. Thakur M. K. (2021). Vitamin B12-folic acid supplementation regulates neuronal immediate early gene expression and improves hippocampal dendritic arborization and memory in old male mice. *Neurochem. Int.* 150:105181. 10.1016/j.neuint.2021.105181 34509560

[B9] Ben Bakrim W. El Bouzidi L. Manouze H. Hafsa J. Sobeh M. BaM’hamed S. (2022). Anti-amnesic effects of withaferin a, a steroidal lactone isolated from *Withania adpressa*, on scopolamine-induced memory impairment in mice. *Arab. J. Chem.* 15:103529. 10.1016/j.arabjc.2021.103529

[B10] Bors W. Heller W. Michel C. Saran M. (1990). Flavonoids as antioxidants: determination of radical-scavenging efficiencies. *Methods Enzymol.* 186 343–355. 10.1016/0076-6879(90)86128-i 2172711

[B11] Brglez Mojzer E. Knez Hrnčič M. Škerget M. Knez Ž Bren U. (2016). Polyphenols: extraction methods, antioxidative action, bioavailability and anticarcinogenic effects. *Molecules* 21:901. 10.3390/molecules21070901 27409600 PMC6273793

[B12] Cabral-Costa J. V. Kowaltowski A. J. (2020). Neurological disorders and mitochondria. *Mol. Aspects Med.* 71:100826. 10.1016/j.mam.2019.10.003 31630771

[B13] Castelli V. Benedetti E. Antonosante A. Catanesi M. Pitari G. Ippoliti R. (2019). Neuronal cells rearrangement during aging and neurodegenerative disease: metabolism, oxidative stress and organelles dynamic. *Front. Mol. Neurosci.* 12:132. 10.3389/fnmol.2019.00132 31191244 PMC6546816

[B14] Catalá A. Díaz M. (2016). Editorial: impact of lipid peroxidation on the physiology and pathophysiology of cell membranes. *Front. Physiol.* 7:423. 10.3389/fphys.2016.00423 27713704 PMC5031777

[B15] Chance B. Sies H. Boveris A. (1979). Hydroperoxide metabolism in mammalian organs. *Physiol. Rev.* 59 527–605. 10.1152/physrev.1979.59.3.527 37532

[B16] Chen Z. Y. Chan P. T. Ho K. Y. Fung K. P. Wang J. (1996). Antioxidant activity of natural flavonoids is governed by number and location of their aromatic hydroxyl groups. *Chem. Phys. Lipids* 79 157–163. 10.1016/0009-3084(96)02523-6 8640902

[B17] Chia N. Wang L. Lu X. Senut M. C. Brenner C. Ruden D. M. (2011). Hypothesis: environmental regulation of 5-hydroxymethylcytosine by oxidative stress. *Epigenetics* 6 853–856. 10.4161/epi.6.7.16461 21617369

[B18] Cvejic J. M. Djekic S. V. Petrovic A. V. Atanackovic M. T. Jovic S. M. Brceski I. D. (2010). Determination of trans- and cis-resveratrol in Serbian commercial wines. *J. Chromatogr. Sci.* 48 229–234. 10.1093/chromsci/48.3.229 20223091

[B19] Dai J. Mumper R. J. (2010). Plant phenolics: extraction, analysis and their antioxidant and anticancer properties. *Molecules* 15 7313–7352. 10.3390/molecules15107313 20966876 PMC6259146

[B20] Dalle-Donne I. Rossi R. Giustarini D. Milzani A. Colombo R. (2003). Protein carbonyl groups as biomarkers of oxidative stress. *Clin. Chim. Acta* 329 23–38. 10.1016/s0009-8981(03)00003-2 12589963

[B21] Davies K. J. Delsignore M. E. Lin S. W. (1987). Protein damage and degradation by oxygen radicals. II. Modification of amino acids. *J. Biol. Chem.* 262 9902–9907. 3036876

[B22] de Whalley C. V. Rankin S. M. Hoult J. R. Jessup W. Leake D. S. (1990). Flavonoids inhibit the oxidative modification of low-density lipoproteins by macrophages. *Biochem. Pharmacol.* 39 1743–1750. 10.1016/0006-2952(90)90120-a 2344371

[B23] Dei R. Takeda A. Niwa H. Li M. Nakagomi Y. Watanabe M. (2002). Lipid peroxidation and advanced glycation end products in the brain in normal aging and in Alzheimer’s disease. *Acta Neuropathol.* 104 113–122. 10.1007/s00401-002-0523-y 12111353

[B24] Drazic A. Winter J. (2014). The physiological role of reversible methionine oxidation. *Biochim. Biophys. Acta* 1844 1367–1382. 10.1016/j.bbapap.2014.01.001 24418392

[B25] EFSA Scientific Committee Hardy A. Benford D. Halldorsson T. Jeger M. J. Knutsen H. K. (2011). Scientific opinion on guidance on the risk assessment of the application of nanoscience and nanotechnologies in the food and feed chain. *EFSA J.* 9:2140. 10.2903/j.efsa.2011.2140 PMC7009542 32625968

[B26] ELBini-Dhouib I. Doghri R. Ellefi A. Degrach I. Srairi-Abid N. Gati A. (2021). Curcumin attenuated neurotoxicity in sporadic animal model of Alzheimer’s disease. *Molecules* 26:3011. 10.3390/molecules26103011 34070220 PMC8158738

[B27] el-Sayed M. M. Abdel-Hameed E. Ahmed W. S. el-Wakil E. A. (2008). Non-phenolic antioxidant compounds from *Buddleja asiatica*. *Z. Naturforsc. C J. Biosci.* 63 483–491. 10.1515/znc-2008-7-803 18810989

[B28] Farout L. Friguet B. (2006). Proteasome function in aging and oxidative stress: implications in protein maintenance failure. *Antioxid. Redox Signal.* 8 205–216. 10.1089/ars.2006.8.205 16487054

[B29] Floyd R. A. Hensley K. (2002). Oxidative stress in brain aging. Implications for therapeutics of neurodegenerative diseases. *Neurobiol. Aging* 23 795–807. 10.1016/s0197-4580(02)00019-2 12392783

[B30] Foti M. C. Amorati R. (2009). Non-phenolic radical-trapping antioxidants. *J. Pharm. Pharmacol.* 61 1435–1448. 10.1211/jpp/61.11.0002 19903368

[B31] Fraga C. G. Croft K. D. Kennedy D. O. Tomás-Barberán F. A. (2019). The effects of polyphenols and other bioactives on human health. *Food Funct.* 10 514–528. 10.1039/c8fo01997e 30746536

[B32] Fukui K. Omoi N. O. Hayasaka T. Shinnkai T. Suzuki S. Abe K. (2002). Cognitive impairment of rats caused by oxidative stress and aging, and its prevention by vitamin E. *Ann. N.Y. Acad. Sci.* 959 275–284. 10.1111/j.1749-6632.2002.tb02099.x 11976202

[B33] Ganesan P. Ko H. Kim I. Choi D. K. (2015). Recent trends in the development of nanophytobioactive compounds and delivery systems for their possible role in reducing oxidative stress in Parkinson’s disease models. *Int. J. Nanomed.* 10 6757–6772. 10.2147/IJN.S93918 26604750 PMC4631432

[B34] Gao Y. Fu R. Wang J. Yang X. Wen L. Feng J. (2018). Resveratrol mitigates the oxidative stress mediated by hypoxic-ischemic brain injury in neonatal rats via Nrf2/HO-1 pathway. *Pharm. Biol.* 56 440–449. 10.1080/13880209.2018.1502326 30460866 PMC6249550

[B35] Gemma C. Vila J. Bachstetter A. Bickford P. C. (2007). “Oxidative stress and the aging brain: from theory to prevention,” in *Brain Aging: Models, Methods, and Mechanisms*, ed. Riddle D. R. (Boca Raton, FL: CRC Press). 21204345

[B36] Gentile F. Arcaro A. Pizzimenti S. Daga M. Cetrangolo G. P. Dianzani C. (2017). DNA damage by lipid peroxidation products: implications in cancer, inflammation and autoimmunity. *AIMS Genet.* 4 103–137. 10.3934/genet.2017.2.103 31435505 PMC6690246

[B37] Giaconia M. A. Ramos S. P. Pereira C. F. Lemes A. C. De Rosso V. V. Cavalcante Braga A. R. (2020). Overcoming restrictions of bioactive compounds biological effects in food using nanometer-sized structures. *Food Hydrocoll.* 107:105939. 10.1016/j.foodhyd.2020.105939

[B38] Gorni D. Finco A. (2020). Oxidative stress in elderly population: a prevention screening study. *Aging Med.* 3 205–213. 10.1002/agm2.12121 33103041 PMC7574639

[B39] Grimm A. Eckert A. (2017). Brain aging and neurodegeneration: from a mitochondrial point of view. *J. Neurochem.* 143 418–431. 10.1111/jnc.14037 28397282 PMC5724505

[B40] Grimm S. Hoehn A. Davies K. J. Grune T. (2011). Protein oxidative modifications in the ageing brain: consequence for the onset of neurodegenerative disease. *Free Radic. Res.* 45 73–88. 10.3109/10715762.2010.512040 20815785 PMC3675897

[B41] Gu X. Sun J. Li S. Wu X. Li L. (2013). Oxidative stress induces DNA demethylation and histone acetylation in SH-SY5Y cells: potential epigenetic mechanisms in gene transcription in Aβ production. *Neurobiol. Aging* 34 1069–1079. 10.1016/j.neurobiolaging.2012.10.013 23141413

[B42] Gülçin I. (2010). Antioxidant properties of resveratrol: a structure–activity insight. *Innov. Food Sci. Emerg. Technol.* 11 210–218. 10.1016/j.ifset.2009.07.002

[B43] Haddadi M. Jahromi S. R. Sagar B. K. Patil R. K. Shivanandappa T. Ramesh S. R. (2014). Brain aging, memory impairment and oxidative stress: a study in *Drosophila melanogaster*. *Behav. Brain Res.* 259 60–69. 10.1016/j.bbr.2013.10.036 24183945

[B44] Hano C. Tungmunnithum D. (2020). Plant polyphenols, more than just simple natural antioxidants: oxidative stress, aging and age-related diseases. *Medicines* 7:26. 10.3390/medicines7050026 32397520 PMC7281114

[B45] Harman D. (1956). Aging: a theory based on free radical and radiation chemistry. *J. Gerontol.* 11 298–300. 10.1093/geronj/11.3.298 13332224

[B46] Harman D. (1972). The biologic clock: the mitochondria? *J. Am. Geriatr. Soc.* 20 145–147. 10.1111/j.1532-5415.1972.tb00787.x 5016631

[B47] Harman D. (1992). Free radical theory of aging. *Mutat. Res.* 275 257–266. 10.1016/0921-8734(92)90030-s 1383768

[B48] Harman D. (2006). Free radical theory of aging: an update: increasing the functional life span. *Ann. N.Y. Acad. Sci.* 1067 10–21. 10.1196/annals.1354.003 16803965

[B49] Head E. (2009). Oxidative damage and cognitive dysfunction: antioxidant treatments to promote healthy brain aging. *Neurochem. Res.* 34 670–678. 10.1007/s11064-008-9808-4 18683046 PMC4392815

[B50] Hu D. Serrano F. Oury T. D. Klann E. (2006). Aging-dependent alterations in synaptic plasticity and memory in mice that overexpress extracellular superoxide dismutase. *J. Neurosci.* 26 3933–3941. 10.1523/JNEUROSCI.5566-05.2006 16611809 PMC6673899

[B51] Huang T. C. Lu K. T. Wo Y. Y. Wu Y. J. Yang Y. L. (2011). Resveratrol protects rats from Aβ-induced neurotoxicity by the reduction of iNOS expression and lipid peroxidation. *PLoS One* 6:e29102. 10.1371/journal.pone.0029102 22220203 PMC3248406

[B52] Huang Y. H. Zhang Q. H. (2010). Genistein reduced the neural apoptosis in the brain of ovariectomised rats by modulating mitochondrial oxidative stress. *Br. J. Nutr.* 104 1297–1303. 10.1017/S0007114510002291 20579403

[B53] Ionescu-Tucker A. Cotman C. W. (2021). Emerging roles of oxidative stress in brain aging and Alzheimer’s disease. *Neurobiol. Aging* 107 86–95. 10.1016/j.neurobiolaging.2021.07.014 34416493

[B54] Jovanovic S. V. Steenken S. Boone C. W. Simic M. G. (1999). H-atom transfer is a preferred antioxidant mechanism of curcumin. *J. Am. Chem. Soc.* 121 9677–9681.

[B55] Jové M. Pradas I. Dominguez-Gonzalez M. Ferrer I. Pamplona R. (2019). Lipids and lipoxidation in human brain aging. mitochondrial ATP-synthase as a key lipoxidation target. *Redox Biol.* 23:101082. 10.1016/j.redox.2018.101082 30635167 PMC6859548

[B56] Kamat P. K. Kalani A. Rai S. Swarnkar S. Tota S. Nath C. (2016). Mechanism of oxidative stress and synapse dysfunction in the pathogenesis of Alzheimer’s disease: understanding the therapeutics strategies. *Mol. Neurobiol.* 53 648–661. 10.1007/s12035-014-9053-6 25511446 PMC4470891

[B57] Kandlur A. Satyamoorthy K. Gangadharan G. (2020). Oxidative stress in cognitive and epigenetic aging: a retrospective glance. *Front. Mol. Neurosci.* 13:41. 10.3389/fnmol.2020.00041 32256315 PMC7093495

[B58] Kelmer Sacramento E. Kirkpatrick J. M. Mazzetto M. Baumgart M. Bartolome A. Di Sanzo S. (2020). Reduced proteasome activity in the aging brain results in ribosome stoichiometry loss and aggregation. *Mol. Syst. Biol.* 16:e9596. 10.15252/msb.20209596 32558274 PMC7301280

[B59] Konar A. Shah N. Singh R. Saxena N. Kaul S. C. Wadhwa R. (2011). Protective role of ashwagandha leaf extract and its component withanone on scopolamine-induced changes in the brain and brain-derived cells. *PLoS One* 6:e27265. 10.1371/journal.pone.0027265 22096544 PMC3214041

[B60] Kowald A. Kirkwood T. B. (2000). Accumulation of defective mitochondria through delayed degradation of damaged organelles and its possible role in the ageing of post-mitotic and dividing cells. *J. Theor. Biol.* 202 145–160. 10.1006/jtbi.1999.1046 10640434

[B61] Kregel K. C. Zhang H. J. (2007). An integrated view of oxidative stress in aging: basic mechanisms, functional effects, and pathological considerations. *Am. J. Physiol. Regul. Integr. Comp. Physiol.* 292 R18–R36. 10.1152/ajpregu.00327.2006 16917020

[B62] Kumar A. Naidu P. S. Seghal N. Padi S. S. (2007). Neuroprotective effects of resveratrol against intracerebroventricular colchicine-induced cognitive impairment and oxidative stress in rats. *Pharmacology* 79 17–26. 10.1159/000097511 17135773

[B63] Lambeth J. D. (2004). NOX enzymes and the biology of reactive oxygen. *Nat. Rev. Immunol.* 4 181–189. 10.1038/nri1312 15039755

[B64] Lee J. Kim Y. S. Kim E. Kim Y. Kim Y. (2020). Curcumin and hesperetin attenuate D-galactose-induced brain senescence in vitro and in vivo. *Nutr. Res. Pract.* 14 438–452. 10.4162/nrp.2020.14.5.438 33029285 PMC7520561

[B65] Lee S. Jeong S. Y. Lim W. C. Kim S. Park Y. Y. Sun X. (2007). Mitochondrial fission and fusion mediators, hFis1 and OPA1, modulate cellular senescence. *J. Biol. Chem.* 282 22977–22983. 10.1074/jbc.M700679200 17545159

[B66] Lei S. Wu S. Wang G. Li B. Liu B. Lei X. (2021). Pinoresinol diglucoside attenuates neuroinflammation, apoptosis and oxidative stress in a mice model with Alzheimer’s disease. *Neuroreport* 32 259–267. 10.1097/WNR.0000000000001583 33470758

[B67] Leonard S. S. Xia C. Jiang B. H. Stinefelt B. Klandorf H. Harris G. K. (2003). Resveratrol scavenges reactive oxygen species and effects radical-induced cellular responses. *Biochem. Biophys. Res. Commun.* 309 1017–1026. 10.1016/j.bbrc.2003.08.105 13679076

[B68] Loeffler D. A. (2019). Influence of normal aging on brain autophagy: a complex scenario. *Front. Aging Neurosci.* 11:49. 10.3389/fnagi.2019.00049 30914945 PMC6421305

[B69] Lu C. Wang Y. Xu T. Li Q. Wang D. Zhang L. (2018). Genistein ameliorates scopolamine-induced amnesia in mice through the regulation of the cholinergic neurotransmission, antioxidant system and the ERK/CREB/BDNF signaling. *Front. Pharm.* 9:1153. 10.3389/fphar.2018.01153 30369882 PMC6194227

[B70] Malik P. Mukherjee T. K. (2014). Structure-function elucidation of antioxidative and prooxidative activities of the polyphenolic compound curcumin. *Chin. J. Biol.* 2014:396708. 10.1155/2014/396708

[B71] Manach C. Scalbert A. Morand C. Rémésy C. Jiménez L. (2004). Polyphenols: food sources and bioavailability. *Am. J. Clin. Nutr.* 79 727–747. 10.1093/ajcn/79.5.727 15113710

[B72] Mattson M. P. Gleichmann M. Cheng A. (2008). Mitochondria in neuroplasticity and neurological disorders. *Neuron* 60 748–766. 10.1016/j.neuron.2008.10.010 19081372 PMC2692277

[B73] Mazza G. Miniati E. (1993). *Anthocyanins in Fruits, Vegetables, and Grains.* Boca Raton, FL: CRC Press, 10.1201/9781351069700

[B74] Mecocci P. Boccardi V. Cecchetti R. Bastiani P. Scamosci M. Ruggiero C. (2018). A long journey into aging, brain aging, and Alzheimer’s disease following the oxidative stress tracks. *J. Alzheimers Dis.* 62 1319–1335. 10.3233/JAD-170732 29562533 PMC5870006

[B75] Menze E. T. Esmat A. Tadros M. G. Abdel-Naim A. B. Khalifa A. E. (2015). Genistein improves 3-NPA-induced memory impairment in ovariectomized rats: impact of its antioxidant, anti-inflammatory and acetylcholinesterase modulatory properties. *PLoS One* 10:e0117223. 10.1371/journal.pone.0117223 25675218 PMC4326416

[B76] Mishra E. Thakur M. K. (2022). Alterations in hippocampal mitochondrial dynamics are associated with neurodegeneration and recognition memory decline in old male mice. *Biogerontology* 23 251–271. 10.1007/s10522-022-09960-3 35266060

[B77] Molaei A. Hatami H. Dehghan G. Sadeghian R. Khajehnasiri N. (2020). Synergistic effects of quercetin and regular exercise on the recovery of spatial memory and reduction of parameters of oxidative stress in animal model of Alzheimer’s disease. *EXCLI J.* 19 596–612. 10.17179/excli2019-2082 32483406 PMC7257248

[B78] Montine T. J. Neely M. D. Quinn J. F. Beal M. F. Markesbery W. R. Roberts L. J. (2002). Lipid peroxidation in aging brain and Alzheimer’s disease. *Free Radic. Biol. Med.* 33 620–626. 10.1016/s0891-5849(02)00807-9 12208348

[B79] Mukherjee S. Dugad S. Bhandare R. Pawar N. Jagtap S. Pawar P. K. (2011). Evaluation of comparative free-radical quenching potential of Brahmi (*Bacopa monnieri*) and Mandookparni (*Centella asiatica*). *Ayu* 32 258–264. 10.4103/0974-8520.92549 22408313 PMC3296351

[B80] Nagai T. Yamada K. Kim H. C. Kim Y. S. Noda Y. Imura A. (2003). Cognition impairment in the genetic model of aging klotho gene mutant mice: a role of oxidative stress. *FASEB J.* 17 50–52. 10.1096/fj.02-0448fje 12475907

[B81] Nakagawa T. Itoh M. Ohta K. Hayashi Y. Hayakawa M. Yamada Y. (2016). Improvement of memory recall by quercetin in rodent contextual fear conditioning and human early-stage Alzheimer’s disease patients. *Neuroreport* 27 671–676. 10.1097/WNR.0000000000000594 27145228

[B82] Navarro A. Boveris A. (2010). Brain mitochondrial dysfunction in aging, neurodegeneration, and Parkinson’s disease. *Front. Aging Neurosci.* 2:34. 10.3389/fnagi.2010.00034 20890446 PMC2947925

[B83] Navarro-Cruz A. R. Ramírez Y. Ayala R. Ochoa-Velasco C. Brambila E. Avila-Sosa R. (2017). Effect of chronic administration of resveratrol on cognitive performance during aging process in rats. *Oxid. Med. Cell. Longev.* 2017:8510761. 10.1155/2017/8510761 29163756 PMC5661096

[B84] Norat P. Soldozy S. Sokolowski J. D. Gorick C. M. Kumar J. S. Chae Y. (2020). Mitochondrial dysfunction in neurological disorders: exploring mitochondrial transplantation. *NPJ Regen. Med.* 5:22. 10.1038/s41536-020-00107-x 33298971 PMC7683736

[B85] Oracz J. Nebesny E. Zyzelewicz D. Budryn G. Luzak B. (2020). Bioavailability and metabolism of selected cocoa bioactive compounds: a comprehensive review. *Crit. Rev. Food Sci. Nutr.* 60 1947–1985. 10.1080/10408398.2019.1619160 31124371

[B86] Pamplona R. (2008). Membrane phospholipids, lipoxidative damage and molecular integrity: a causal role in aging and longevity. *Biochim. Biophys. Acta* 1777 1249–1262. 10.1016/j.bbabio.2008.07.003 18721793

[B87] Paradies G. Petrosillo G. Paradies V. Ruggiero F. M. (2011). Mitochondrial dysfunction in brain aging: role of oxidative stress and cardiolipin. *Neurochem. Int.* 58 447–457. 10.1016/j.neuint.2010.12.016 21215780

[B88] Pesce M. Tatangelo R. La Fratta I. Rizzuto A. Campagna G. Turli C. (2018). Aging-related oxidative stress: positive effect of memory training. *Neuroscience* 370 246–255. 10.1016/j.neuroscience.2017.09.046 28987510

[B89] Pierzynowska K. Gaffke L. Hać A. Mantej J. Niedziałek N. Brokowska J. (2018). Correction of Huntington’s disease phenotype by genistein-induced autophagy in the cellular model. *Neuromolecular Med.* 20 112–123. 10.1007/s12017-018-8482-1 29435951 PMC5834590

[B90] Qian Y. Cao L. Guan T. Chen L. Xin H. Li Y. (2015). Protection by genistein on cortical neurons against oxidative stress injury via inhibition of NF-kappaB, JNK and ERK signaling pathway. *Pharm. Biol.* 53 1124–1132. 10.3109/13880209.2014.962057 25715966

[B91] Quideau S. Deffieux D. Douat-Casassus C. Pouységu L. (2011). Plant polyphenols: chemical properties, biological activities, and synthesis. *Angew. Chem.* 50 586–621. 10.1002/anie.201000044 21226137

[B92] Rajan V. K. Muraleedharan K. (2017). A computational investigation on the structure, global parameters and antioxidant capacity of a polyphenol, gallic acid. *Food Chem.* 220 93–99. 10.1016/j.foodchem.2016.09.178 27855941

[B93] Rasouli H. Farzaei M. H. Khodarahmi R. (2017). Polyphenols and their benefits. *Int. J. Food Prop.* 20 1700–1741. 10.1080/10942912.2017.1354017

[B94] Reeg S. Grune T. (2015). Protein oxidation in aging: does it play a role in aging progression? *Antioxid. Redox Signal.* 23 239–255. 10.1089/ars.2014.6062 25178482 PMC4507125

[B95] Rege S. D. Geetha T. Griffin G. D. Broderick T. L. Babu J. R. (2014). Neuroprotective effects of resveratrol in Alzheimer disease pathology. *Front. Aging Neurosci.* 6:218. 10.3389/fnagi.2014.00218 25309423 PMC4161050

[B96] Richter C. Park J. W. Ames B. N. (1988). Normal oxidative damage to mitochondrial and nuclear DNA is extensive. *Proc. Natl. Acad. Sci. U.S.A.* 85 6465–6467. 10.1073/pnas.85.17.6465 3413108 PMC281993

[B97] Rodrigues Siqueira I. Fochesatto C. da Silva Torres I. L. Dalmaz C. Alexandre Netto C. (2005). Aging affects oxidative state in hippocampus, hypothalamus and adrenal glands of Wistar rats. *Life Sci.* 78 271–278. 10.1016/j.lfs.2005.04.044 16112138

[B98] Rutten B. P. Schmitz C. Gerlach O. H. Oyen H. M. de Mesquita E. B. Steinbusch H. W. (2007). The aging brain: accumulation of DNA damage or neuron loss? *Neurobiol. Aging* 28 91–98. 10.1016/j.neurobiolaging.2005.10.019 16338029

[B99] Sarker M. R. Franks S. F. (2018). Efficacy of curcumin for age-associated cognitive decline: a narrative review of preclinical and clinical studies. *Geroscience* 40 73–95. 10.1007/s11357-018-0017-z 29679204 PMC5964053

[B100] Setchell K. D. Brown N. M. Zimmer-Nechemias L. Brashear W. T. Wolfe B. E. Kirschner A. S. (2002). Evidence for lack of absorption of soy isoflavone glycosides in humans, supporting the crucial role of intestinal metabolism for bioavailability. *Am. J. Clin. Nutr.* 76 447–453. 10.1093/ajcn/76.2.447 12145021

[B101] Shalini V. T. Neelakanta S. J. Sriranjini J. S. (2021). Neuroprotection with *Bacopa monnieri*-a review of experimental evidence. *Mol. Biol. Rep.* 48 2653–2668. 10.1007/s11033-021-06236-w 33675463

[B102] Shanbhag N. M. Evans M. D. Mao W. Nana A. L. Seeley W. W. Adame A. (2019). Early neuronal accumulation of DNA double strand breaks in Alzheimer’s disease. *Acta Neuropathol. Commun.* 7:77. 10.1186/s40478-019-0723-5 31101070 PMC6524256

[B103] Singh P. Konar A. Kumar A. Srivas S. Thakur M. K. (2015). Hippocampal chromatin-modifying enzymes are pivotal for scopolamine-induced synaptic plasticity gene expression changes and memory impairment. *J. Neurochem.* 134 642–651. 10.1111/jnc.13171 25982413

[B104] Singh P. Sivanandam T. M. Konar A. Thakur M. K. (2021). Role of nutraceuticals in cognition during aging and related disorders. *Neurochem. Int.* 143:104928. 10.1016/j.neuint.2020.104928 33285273

[B105] Smith C. D. Carney J. M. Starke-Reed P. E. Oliver C. N. Stadtman E. R. Floyd R. A. (1991). Excess brain protein oxidation and enzyme dysfunction in normal aging and in Alzheimer disease. *Proc. Natl. Acad. Sci. U.S.A.* 88 10540–10543. 10.1073/pnas.88.23.10540 1683703 PMC52964

[B106] Somparn P. Phisalaphong C. Nakornchai S. Unchern S. Morales N. P. (2007). Comparative antioxidant activities of curcumin and its demethoxy and hydrogenated derivatives. *Biol. Pharm. Bull.* 30 74–78. 10.1248/bpb.30.74 17202663

[B107] Soquetta M. B. Terra L. M. Bastos C. P. (2018). Green technologies for the extraction of bioactive compounds in fruits and vegetables. *CYTA J. Food* 16 400–412. 10.1080/19476337.2017.1411978

[B108] Srivas S. Baghel M. S. Singh P. Thakur M. K. (2020). “Neurodegeneration during aging: role of oxidative stress through epigenetic modifications,” in *Models, Molecules and Mechanisms in Biogerontology*, ed. Rath P. C. (New York, NY: Springer press), 43–55.

[B109] Stadtman E. R. (1992). Protein oxidation and aging. *Science* 257 1220–1224. 10.1126/science.1355616 1355616

[B110] Stadtman E. R. (2004). Role of oxidant species in aging. *Curr. Med. Chem.* 11 1105–1112. 10.2174/0929867043365341 15134509

[B111] Staniek K. Nohl H. (2000). Are mitochondria a permanent source of reactive oxygen species? *Biochim. Biophys. Acta* 1460 268–275. 10.1016/s0005-2728(00)00152-3 11106768

[B112] Stojanović S. Sprinz H. Brede O. (2001). Efficiency and mechanism of the antioxidant action of trans-resveratrol and its analogues in the radical liposome oxidation. *Arch. Biochem. Biophys.* 391 79–89. 10.1006/abbi.2001.2388 11414688

[B113] St-Pierre J. Buckingham J. A. Roebuck S. J. Brand M. D. (2002). Topology of superoxide production from different sites in the mitochondrial electron transport chain. *J. Biol. Chem.* 277 44784–44790. 10.1074/jbc.M207217200 12237311

[B114] Suckow B. K. Suckow M. A. (2006). Lifespan extension by the antioxidant curcumin in *Drosophila melanogaster*. *Int. J. Biomed. Sci.* 2 402–405. 23675008 PMC3614642

[B115] Sultana R. Perluigi M. Butterfield D. A. (2013). Lipid peroxidation triggers neurodegeneration: a redox proteomics view into the Alzheimer disease brain. *Free Radic. Biol. Med.* 62 157–169. 10.1016/j.freeradbiomed.2012.09.027 23044265 PMC3573239

[B116] Sun Q. Wedick N. M. Pan A. Townsend M. K. Cassidy A. Franke A. A. (2014). Gut microbiota metabolites of dietary lignans and risk of type 2 diabetes: a prospective investigation in two cohorts of U.S. women. *Diabetes Care* 37 1287–1295. 10.2337/dc13-2513 24550220 PMC3994932

[B117] Terman A. Kurz T. Navratil M. Arriaga E. A. Brunk U. T. (2010). Mitochondrial turnover and aging of long-lived postmitotic cells: the mitochondrial-lysosomal axis theory of aging. *Antioxid. Redox Signal.* 12 503–535. 10.1089/ars.2009.2598 19650712 PMC2861545

[B118] Thakur M. K. Konar A. Kumar D. Baghel M. S. Singh P. (2017). “Recovery of age-related memory loss: hopes and challenges,” in *Topics in Biomedical Gerontology*, eds Prasad S. Rath P. C. Sharma R. (New York, NY: Springer Press), 267–278.

[B119] Thanan R. Oikawa S. Hiraku Y. Ohnishi S. Ma N. Pinlaor S. (2014). Oxidative stress and its significant roles in neurodegenerative diseases and cancer. *Int. J. Mol. Sci.* 16 193–217. 10.3390/ijms16010193 25547488 PMC4307243

[B120] Tsai T. H. (2005). Concurrent measurement of unbound genistein in the blood, brain and bile of anesthetized rats using micro dialysis and its pharmacokinetic application. *J. Chromatogr.* 1073 317–322. 10.1016/j.chroma.2004.10.048 15909536

[B121] Tsao R. (2010). Chemistry and biochemistry of dietary polyphenols. *Nutrients* 2 1231–1246. 10.3390/nu2121231 22254006 PMC3257627

[B122] Túnez I. Tasset I. Pérez-De La Cruz V. Santamaría A. (2010). 3-Nitropropionic acid as a tool to study the mechanisms involved in Huntington’s disease: past, present and future. *Molecules* 15 878–916. 10.3390/molecules15020878 20335954 PMC6263191

[B123] Tzeng C. W. Yen F. L. Wu T. H. Ko H. H. Lee C. W. Tzeng W. S. (2011). Enhancement of dissolution and antioxidant activity of kaempferol using a nanoparticle engineering process. *J. Agric. Food Chem.* 59 5073–5080. 10.1021/jf200354y 21417334

[B124] Upadhyay R. K. (2014). Drug delivery systems, CNS protection, and the blood brain barrier. *Biomed Res. Int.* 2014:869269. 10.1155/2014/869269 25136634 PMC4127280

[B125] Viña J. Sastre J. Pallardó F. Borrás C. (2003). Mitochondrial theory of aging: importance to explain why females live longer than males. *Antioxid. Redox Signal.* 5 549–556. 10.1089/152308603770310194 14580309

[B126] Wang B. Yang Q. Sun Y. Y. Xing Y. F. Wang Y. B. Lu X. T. (2014). Resveratrol-enhanced autophagic flux ameliorates myocardial oxidative stress injury in diabetic mice. *J. Cell. Mol. Med.* 18 1599–1611. 10.1111/jcmm.12312 24889822 PMC4190906

[B127] Yoritaka A. Hattori N. Uchida K. Tanaka M. Stadtman E. R. Mizuno Y. (1996). Immunohistochemical detection of 4-hydroxynonenal protein adducts in Parkinson disease. *Proc. Natl. Acad. Sci. U.S.A.* 93 2696–2701. 10.1073/pnas.93.7.2696 8610103 PMC39693

[B128] Youssef F. S. Menze E. T. Ashour M. L. (2020). A potent lignan from prunes alleviates inflammation and oxidative stress in Lithium/Pilocarpine-induced epileptic seizures in rats. *Antioxidants* 9:575. 10.3390/antiox9070575 32630680 PMC7402155

[B129] Zarkovic K. (2003). 4-Hydroxynonenal and neurodegenerative diseases. *Mol. Aspects Med.* 24 293–303. 10.1016/s0098-2997(03)00024-4 12893007

[B130] Zeb A. (2020). Concept, mechanism, and applications of phenolic antioxidants in foods. *J. Food Biochem.* 44:e13394. 10.1111/jfbc.13394 32691460

[B131] Zeng H. Chen Q. Zhao B. (2004). Genistein ameliorates beta-amyloid peptide (25-35)-induced hippocampal neuronal apoptosis. *Free Radic. Biol. Med.* 36 180–188. 10.1016/j.freeradbiomed.2003.10.018 14744630

[B132] Zheng Q. Huang T. Zhang L. Zhou Y. Luo H. Xu H. (2016). Dysregulation of ubiquitin-proteasome system in neurodegenerative diseases. *Front. Aging Neurosci.* 8:303. 10.3389/fnagi.2016.00303 28018215 PMC5156861

